# Tailored duration of adjuvant trastuzumab for human epidermal growth factor receptor 2-positive breast cancer

**DOI:** 10.1038/s41698-020-00128-1

**Published:** 2020-08-05

**Authors:** Ke-Da Yu, Xin Wang, Wan-Kun Chen, Lei Fan, Miao Mo, Han Chen

**Affiliations:** 1 Department of Breast Surgery, Fudan University Shanghai Cancer Center, Shanghai Medical College, Fudan University, Shanghai, 200032 China; 2 Department of Anesthesiology, Fudan University Shanghai Cancer Center, Shanghai Medical College, Fudan University, Shanghai, 200032 China; 3 Department of Anesthesiology, Zhongshan Hospital, Fudan University, Shanghai, 200032 China; 4 Department of Cancer Prevention and Cancer Statistics, Fudan University Shanghai Cancer Center, Shanghai Medical College, Fudan University, Shanghai, 200032 China; 5 Department of Ophthalmology, Eye, Ear, Nose and Throat Hospital, Fudan University, Shanghai, 200031 China

**Keywords:** Breast cancer, Outcomes research

## Abstract

We assumed that the effect of adjuvant trastuzumab on survival is mediated by the treatment time and we conducted this trial-level meta-regression to determine the appropriate length of treatment. Twelve adjuvant trastuzumab trials (from January 2000 to June 2019, consisting of 20,271 patients) were included. We considered 12-month trastuzumab treatment as the standard. The primary study endpoint was disease-free survival (DFS). By quantifying the relationship between shortened treatment time (month) and altered recurrence risk (expressed as hazard ratio), we found the regression coefficient *β* was 0.05 (95% confidence interval: 0.02–0.08, *P* = 0.002), indicating the recurrence risk would increase 5.1% for each month that treatment was shortened. Accordingly, 3, 6, and 9-month reductions in treatment time resulted in 16%, 35%, and 57% increases in recurrence risk, respectively. We revealed a significant linear association between shortened treatment time of trastuzumab and recurrence risk. The clinical duration of adjuvant trastuzumab should be tailored.

## Introduction

At least four large, randomized, controlled clinical trials have demonstrated that women with early human epidermal growth factor receptor 2 (HER2)-positive breast cancer receiving 12-month adjuvant trastuzumab show improved disease-free survival (DFS) compared with those who did not receive this treatment^1^. Overall survival (OS) improved in agreement with DFS^2^. Although a longer duration of trastuzumab treatment of 24 months seems to add toxicity without additional survival benefit^3^, the shorter duration of a 9-week trastuzumab regimen only exhibited a borderline significant improvement in DFS^4^. Thereafter, a number of clinical trials were conducted to assess the efficacy and toxicity of shorter durations of adjuvant trastuzumab compared with the standard 12-month duration. All such trials were designed as non-inferiority studies, because shorter treatment time with equivalent efficacy would help reducing both costs and treatment toxicities.

However, interpretation of a non-inferiority trial is difficult, even more so for a negative non-inferiority trial. Five non-inferiority trials were conducted on the topic and the conclusions were discordant. PHARE^5,6^, HORG^7^, SOLD^8^, and Short-HER^9^ suggest that the 12-month duration of trastuzumab should remain the gold standard. In contrast, PERSEPHONE demonstrated that a 6-month trastuzumab treatment was non-inferior to a 12-month duration but with reduced toxicity^10^. Given these discrepancies, a meta-analysis of all relevant studies might be more informative. However, as treatment time varied among these trials (from 9 weeks to 12 months), the results of a meta-analysis are still difficult to interpret regarding what a hazardous treatment duration is and how long is sufficiently effective.

As the reduction in duration of trastuzumab treatment varied among trials, we assumed that the effect on DFS was mediated by the treatment time and focused on estimating the hazard ratio (HR) for a given degree of time reduction (e.g., every month). To assess the effect of treatment time of adjuvant trastuzumab on survival outcomes, we conducted the present trial-level meta-regression analysis of randomized, controlled trials, to determine the appropriate length of trastuzumab treatment.

## Results

### Trials included in the analysis

**Fig. 1:**
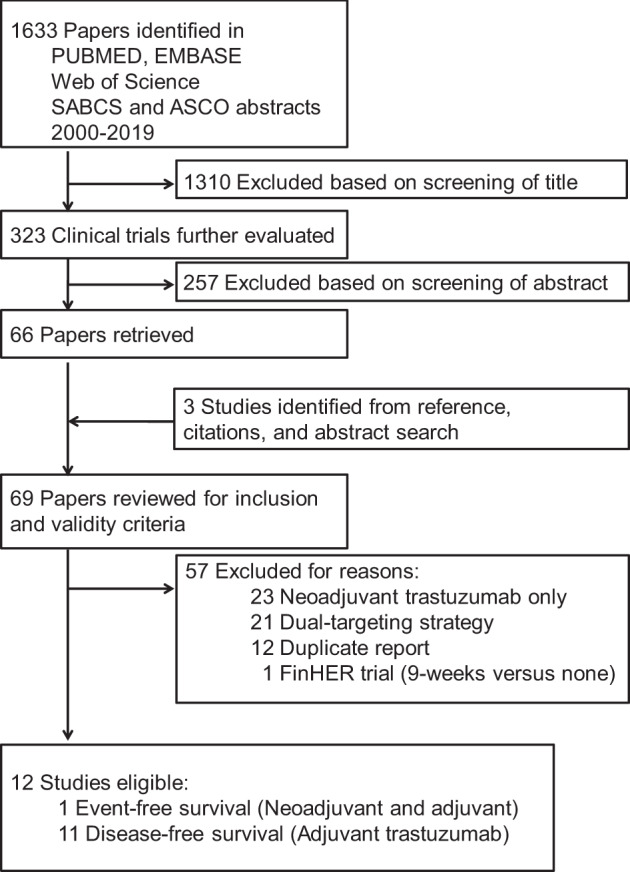
Flow diagram of the literature search process. Literature search results.

A flow diagram of the literature search is shown in Fig. 1. We identified 1633 citations. Among a total of 69 highly relevant randomized studies, we identified 12 eligible trials including 20,271 patients. The primary characteristics of included trials are listed in Table 1. Among the 12 trials, 5 studies compared the standard 12-month trastuzumab treatment with shorter treatments in a non-inferiority setting and 7 compared the 12-month trastuzumab treatment with no trastuzumab in a superiority design.

**Table 1:** Summary of included studies.

Trial	Patient number	Trial design	Country or region	Median age (years)	Node negative	Hormone receptor positive	Adjuvant chemotherapy^a^	Non-inferiority margin of HR	HR with 95% CI	Shortened time (month)	Endpoint	Median follow-up (year)
PHARE^6^	1691	Non-inferiority	France	55	55%	61%	A + T 73%; A 15%; T 11%	1.15	1.08 (0.93–1.25)	6	DFS	7.5
HORG^7^	481	Non-inferiority	Greece	55	21%	67%	Unspecified	1.53	1.57 (0.86–2.10)	6	DFS	3
Short-HER^9^	1254	Non-inferiority	Italy	55	54%	68%	A + T 100%	1.29	1.13 (0.85–1.49)^b^	9.9	DFS	6
SOLD^8^	2174	Non-inferiority	Finland	56	60%	66%	A + T 100%	1.3	1.39 (1.08–1.80)^b^	9.9	DFS	5.2
PERSEPHONE^10^	4088	Non-inferiority	UK	56	58%	69%	A + T 48%; A 42%; T 10%	1.29	1.07 (0.90–1.27)^b^	6	DFS	5.4
E2198^24^	227	Superiority	USA	49	0	62%	Unspecified	NA	1.18 (0.56–2.44)	12	DFS	6.4
NCCTG N9831^12^	1944	Superiority	USA	51	15%	54%	A + T 100%	NA	1.67 (1.47–1.89)	12	DFS	8.4
NSABP B-31^12^	2102	Superiority	USA	49	0	56%	A + T 100%	NA	1.67 (1.47–1.89)	12	DFS	8.4
HERA^25^	3401	Superiority	Global	49	32%	45%	A + T 26%; A 68%	NA	1.45 (1.27–1.69)^c^	12	DFS	8
BCIRG-006^11^	2147	Superiority	USA	48	29%	54%	A + T 100%	NA	1.39 (1.18–1.64)	12	DFS	10.3
PACS04^26^	528	Superiority	Europe	49	0	60%	A + T 47%; A 53%	NA	1.16 (0.82–1.64)	12	DFS	3.9
NOAH^27^	235	Superiority	Global	52	15%	36%	A + T 100%	NA	1.69 (1.11–2.63)	12	EFS	5.4

*CI* confidence interval, *DFS* disease-free survival, *EFS* event-free survival, *HR* hazard ratio, *NA* not applicable.

^a^A, Anthracycline-based; T, Taxane-based; A + T, anthracycline and taxane-containing.

^b^Calculation of the 95% CI using the 90% CI provided in the original paper.

^c^Using the censored analysis in HERA trial. The censored analysis removed the crossover patients’ follow-up, which is a confounding factor, after the start of treatment with trastuzumab.

The BCIRG-006 trial had three comparison arms and the two arms sharing the same chemotherapeutic regimen (doxorubicin and cyclophosphamide followed by docetaxel [AC-T] without trastuzumab vs. the same chemotherapy plus 12-month trastuzumab [AC-TH]) were chosen for analysis^11^. In the E2198 trial, HER2 status was re-assessed based on more contemporary definitions. Considering that trastuzumab is an anti-HER2 treatment and should be strictly administered in HER2-positive populations, we chose the DFS data from the patients centrally retested to be HER2 positive. In the NOAH trial, the study group received the neoadjuvant and adjuvant trastuzumab for 12 months in total, whereas the control group did not. In the PACS04 trial design, the study group should have received 12-month trastuzumab treatment. However, of the 260 patients randomly assigned to the trastuzumab group, 10% did not receive any trastuzumab; of the 234 patients who received at least one dose of trastuzumab, 25% received trastuzumab for <6 months. Therefore, we adjusted the treatment time difference between the study group and control group as approximate to 9.5 months. The 9.5-month treatment was used in the meta-regression instead of 12 months. The NCCTG N9831 and NSABP B-31 trials assessed the efficacy and safety of adding trastuzumab to paclitaxel following doxorubicin and cyclophosphamide. As the two trials were similar in design, it allowed the US Food and Drug Administration to approve a joint efficacy analysis plan that could be executed before the planned analysis of the individual trials. We here used the combined results^12^.

### The relationship between impaired survival and shortened treatment time

In general, a shorter treatment duration (varying from 6 months to 12 months) was associated with a significantly higher risk for breast cancer relapse (HR = 1.32, 95% confidence interval (CI), 1.17–1.49, *P* < 0.0001, Supplementary Fig. S1).

**Fig. 2:**
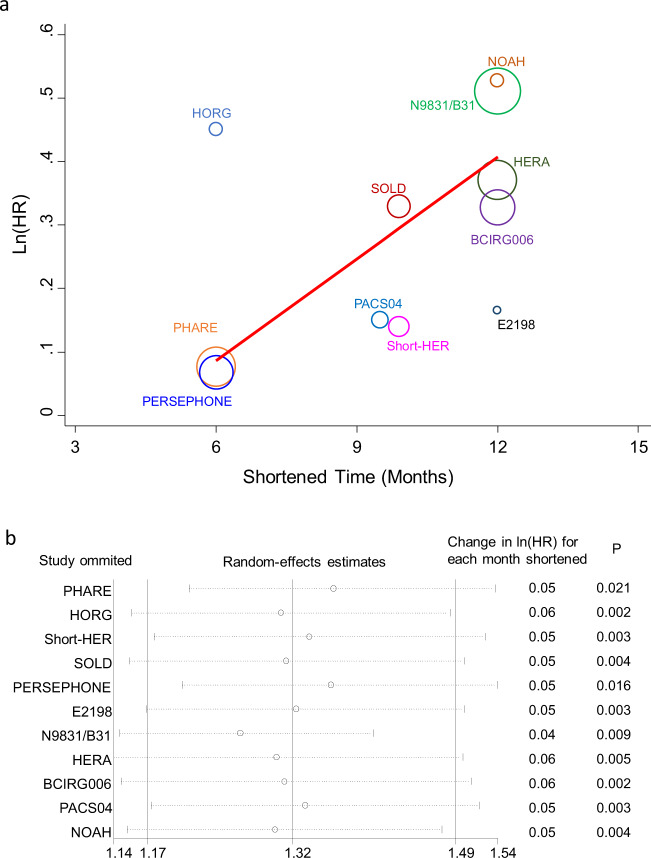
Bubble plot of meta-regression and influence plot of sensitivity analysis. **a** Bubble plot with fitted meta-regression line. Trial-level association between the hazard ratio for disease-free survival and shortened trastuzumab treatment time. The size of the circles in the plot is inversely proportional to the variance of the log odds ratio, so larger circles correspond to larger studies. **b** Sensitivity analysis. Influence of individual studies on the summary effect. The vertical axis indicates the overall HR and the two vertical axes indicate its 95% CI. Every hollow round indicates the pooled HR when the left trial is omitted. The results of meta-regression with *P*-values are also displayed.

We quantified the relationship between shortened treatment time and HR (Fig. 2a). The estimated between-study variance (τ2) was 0.001 and the coefficient of determination (*R*^2^) was 93.9%, indicating 93.9% of variability among the effects on DFS were explained by the shortened treatment time.

The regression coefficient (*β*) of shortened treatment time is 0.05 (95% CI: 0.02–0.08, *P* = 0.002). For each month of reduced trastuzumab, the corresponding HR value is of 1.051 (*e*^0.05^), indicating an increased relative risk in recurrence of 5.1%. Accordingly, 3, 6, and 9-month reductions in treatment time resulted in 16%, 35%, and 57% increases in recurrence risk, respectively (Table 2).

**Table 2:** Shortened adjuvant trastuzumab treatment duration and increased risk of DFS.

Shortened time (months)	Change in ln(HR) (regression coefficient *β*)	Increased relative risk of disease recurrence	Predicted DFS in the APT trial^a^ (%)	Predicted DFS in the N9831/B31 trials^b^ (%)
0	Ref.	0	93	74
1	0.05	5.1%	93	73
3	0.15	16%	92	70
6	0.30	35%	91	66
9	0.45	57%	89	60

^a^In the 12-month trastuzumab group, 7-year DFS of 93% at baseline. None of the trial patients with node-positive disease.

^b^In the 12-month trastuzumab group, 10-year DFS of 74% at baseline. Patients (100%) in B-31 and 86% in N9831 trial with node-positive disease.

Sensitivity analyses were performed by omitting each study yielded similar results (Fig. 2b). There was no evidence of publication bias (degree of asymmetry by Egger’s test *P* = 0.72), with the Begg’s funnel plot shown in Supplementary Fig. S2.

### Exploratory subgroup analyses

Further subgroup analysis was performed by dividing studies according to hormone receptor status, lymph nodes status, and chemotherapeutic regimens (Table 3). The shortened time of trastuzumab was more likely to influence the cancer relapse in patients receiving more aggressive chemotherapy such as anthracycline and taxane-containing regimen (*β* = 0.06, *P* = 0.037), as well as in those with hormone receptor-positive disease (*β* = 0.06, *P* = 0.021).

**Table 3:** Overall and subgroup analyses for shortened time of each month.

Population	Study number	Change in ln(HR) (regression coefficient *β*)	*P*
Overall	12	0.05	0.002
Hormone receptor negative	10	0.04	0.089
Hormone receptor positive	10	0.06	0.021
Node negative	6	0.05	0.20
Node positive	9	0.04	0.083
Chemotherapy-anthracycline and taxane-containing^a^	7	0.06	0.037
Chemotherapy-the others^b^	5	0.04	0.16

^a^Include six trials with 100% of patients using anthracycline and taxane-containing chemotherapy, and one trial (PHARE trial) with 73% of patients using this regimen.

^b^Including anthracycline-based, taxane-based, or unspecified chemotherapy regimens.

## Discussion

In the present meta-regression analysis of randomized controlled trials, we reveal for the first time, to our best knowledge, a significant linear association between shortened trastuzumab treatment time and risk of breast cancer relapse. Our findings imply that a shorter duration of trastuzumab might be considered for patients with lower tumor burden disease (e.g., node negative or small tumor size) but not for those with high-risk disease (e.g., node-positive, larger tumor size, as well as being treated with aggressive chemotherapeutic regimens).

A large meta-analysis of pivotal clinical trials has demonstrated improvements in both DFS and OS with 12-month adjuvant trastuzumab treatment of HER2-positive early breast cancer compared with no use of anti-HER2 treatment^13^. Subsequent trials have examined whether a shorter course of trastuzumab provides a safer, less expensive, and more convenient alternative to 12-month trastuzumab without significantly compromising the risk of cancer recurrence. The FinHER trial had suggested that a shortened duration of trastuzumab could also be effective^4^. The trials evaluating shorter duration of trastuzumab were often appropriately designed as non-inferiority studies. However, the non-inferiority margin varied substantially among trials. Surprisingly, one of the trials accepted up to 53% more adverse survival events with the shorter treatment duration compared with the 12-month treatment as being non-inferior. The upper limits (ULs) of HR for DFS to declare non-inferiority in the 5 included studies were 1.15 (PHARE), 1.53 (HORG), 1.29 (Short-HER), 1.3 (SOLD), and 1.29 (PERSEPHONE). An inappropriate non-inferiority margin might result in different conclusions.

Treatment time is an important parameter influencing treatment outcome. For example, the effect of a delay in time to adjuvant chemotherapy on survival outcomes in breast cancer is a quantitative effect^14^. If a patient is delayed 4 weeks in receiving adjuvant chemotherapy, this patient would have a 15% increased risk of mortality; if delayed 8 weeks, the patient would have a 32% increased risk. Thus far, although a meta-analysis demonstrated that 12-month trastuzumab treatment prolongs OS and DFS in women with early-stage HER2-positive breast cancer compared with shorter durations^15^, there is no study quantifying the association between shortened time and survival outcomes. For the first time, our analysis reveals a linear association between shortened trastuzumab treatment time and risk of breast cancer relapse. For each month of reduced treatment, the ln(HR) increased by 0.05 and the corresponding HR value was 1.051 (*e*^0.05^) with the 95% CIs of 1.02–1.09. In other words, for each reduced month of trastuzumab, there was a relative risk in breast cancer relapse of 5.1%. Similarly, if the shortened treatment time was 3 months, the increased risk was 16% (*e*^0.15^ = 1.16), a 6-month shortened treatment increased the risk by 35%, and a 9-month, by 57%.

Moreover, we re-affirmed the results in different subgroups. The subgroup analyses according to hormone receptor, lymph node status, and chemotherapeutic regimens yielded qualitatively similar results. Notably, the reduced time of trastuzumab was more likely to influence the cancer relapse in patients receiving more aggressive chemotherapy such as anthracycline and taxane-containing regimen. This is plausible because sufficient treatment time of trastuzumab might be important for those with higher HER2-positive tumor burden. Although aggressive chemotherapy might achieve more protection in a general cytotoxic way, anti-HER2-targeted therapy is the key treatment for HER2-positive tumors, because it specifically and directly inhibits the activated HER2 signaling pathway. It has been proven that the adjuvant treatment with trastuzumab-emtansine (T-DM1) in patients with residual HER2-positive disease following neoadjuvant anti-HER2-based treatment, as well as the addition of an anti-HER2 drug such as pertuzumab to trastuzumab, would yield more efficacy compared with adjuvant trastuzumab only^16,17^.

Although relative risk was similar in node-positive subgroup and node-negative subgroup, the absolute risk would be completely different. Therefore, a shorter duration of trastuzumab might be appropriate for some patients with lower-risk disease without concern for diminished survival benefits. For instance, among low-risk patients in the APT trial^18^, who were with node-negative disease and tumor size <3 cm, 6-month reduction of trastuzumab treatment conferred less than a 2% survival rate decrease according to our prediction (Table 2). In contrast, in patients with high-risk disease (100% patients in B-31 and 86% in N9831 had node-positive disease) in the joint N9831/B-31 analysis, a 6-month treatment would result in an absolute 8% DFS benefit loss (from 10-year DFS 74 to 66%) according to our prediction, which is unacceptable, because the total absolute DFS benefit of 12-month trastuzumab was only 11%^12^. We should tailor the duration of adjuvant trastuzumab according to the clinical characteristics of patients. Moreover, we provide the useful information for further non-inferiority clinical trial design. It seems that the non-inferiority limit for the HR should be set at ~1.35 for a 6-month reduction in treatment time.

We acknowledge the following limits in our analysiss. First, we used summary estimates rather than individual patient level data. Second, the follow-up time is heterogeneous among the included studies. The differential median follow-up times should be taken into considerations. The importance of long-term follow-up in trials of HER2-positive breast cancer has been emphasized by several studies in which results have changed over time. The 7-year follow-up in the PHARE trial showed a reduction in HR for disease recurrence compared with the 2-year results^6^. Third, our study relies on the assumption of a log-linear relationship for the effect of reduction time on breast cancer survival. The assumption of linearity to this relationship might sometimes be problematic. For instance, PERSEPHONE showed that survival was similar (HR = 1.07, with 90% CI 0.93–1.24) if they used 6-month trastuzumab^10^. However, our regression results from a combination analysis of all the relevant trials seems to be more accurate and reliable. Fourth, the use of trastuzumab is always combined with chemotherapy. The chemotherapy regiments varied between studies. As the original papers did not provide the chemotherapy subgroup analysis, we could not adjust the confounding effect of chemotherapy in clinical outcome, but we did trial-level subgroup analysis according to different chemotherapy regimens. Finally, in the era of adjuvant dual HER2 targeting, our results could not provide an accurate guidance for the reduction of pertuzumab.

Taken together, our results reveal a significant linear association between shortened trastuzumab treatment duration and risk of DFS events. A shorter duration of trastuzumab treatment might be appropriate for patients with lower-risk disease but unacceptable for those with high-risk disease. We believe that the duration of adjuvant trastuzumab should be tailored according to the risk, as well as toxicity and cost. Further studies to validate and confirm our findings are encouraged.

## Methods

### Selection of studies

A comprehensive search of PUBMED, EMBASE, and Web of Science databases from January 2000 to June 2019 was performed. These dates were chosen, because clinical publications regarding neoadjuvant and/or adjuvant use of trastuzumab likely only occur after year 2000. Key words for searching were as follows: breast, breast carcinoma, breast cancer; HER2, ERBB2, *neu*; trastuzumab, Herceptin; trial, prospective study; adjuvant, neoadjuvant, postoperative, preoperative; and survival, disease-free survival (DFS), event-free survival (EFS), relapse-free survival (RFS), overall survival (OS), prognosis, outcomes. We also manually searched relevant references. Presentations and abstracts from the San Antonio Breast Cancer Symposium and the American Society of Clinical Oncology annual meeting were also searched. To reduce publication bias, all publication types—full-text article, correspondence, and meeting abstract—were eligible. Inclusion criteria were as follows: (1) randomized controlled trial comparing 12-month adjuvant trastuzumab (could be administered before surgery) with shorter durations. Treatment with dual HER2-targeting regimens were ineligible. (2) Outcomes could be presented as DFS, EFS, or RFS. HR with 95% CIs were reported. (3) Studies should be published in English. The systematic review and meta-analysis was designed according to the PRISMA guidelines^19^. The study protocol was approved by the Institutional Ethics Committee of Fudan University Shanghai Cancer Center. For each original trial, we had checked that the trial study protocol was approved by the Institutional Review Board and every participating patient had supplied written informed consent.

### Data extraction and treatment

In this study, we considered “12-month trastuzumab” as the standard treatment. We included clinical trials comparing 12-month trastuzumab with shorter trastuzumab treatment durations in early-stage HER2-positive breast cancer in the (neo)adjuvant setting. The “shorter duration” should be <12 months, including the extreme value of 0 month, i.e., no use of adjuvant trastuzumab.

Data for 24-month use of trastuzumab in the HERA trial^3^ were excluded, because we did not investigate treatment durations longer than 12 months in the present study. Of note, in the FinHER trial design, 9-week trastuzumab treatment was compared with no trastuzumab treatment. This trial was also excluded, because we treated the “12-month” treatment as the standard and all shorter durations were compared with the standard duration.

If the original study provided a 90% CI, we calculated the 95% lower limit (LL) and UL using the follow formulas:

$$95\% \,{\mathrm{LL}} = {{e}}^{\left( {{\mathrm{lnHR}} - 1.96 \ast \left( {\left[ ln{90\% {\mathrm{UL}} - ln90\% {\mathrm{LL}}} \right]/1.64/2} \right)} \right)}$$

$$95\% \,{\mathrm{UL}} = {{e}}^{\left( {{\mathrm{lnHR}} + 1.96 \ast \left( {\left[ {ln90\% {\mathrm{UL}} - ln90\% {\mathrm{LL}}} \right]/1.64/2} \right)} \right)}$$

The SE of ln(HR) was calculated by (ln[95%UL] − ln[95%LL])/1.96/2.

The primary study endpoint was DFS in the adjuvant trials. As DFS is inappropriate in neoadjuvant studies, we considered EFS instead.

Two investigators (Y.K.D. and F.L.) independently assessed studies for inclusion and extracted relevant data. Disagreements were resolved by discussion until consensus by all authors was reached. If necessary, the corresponding author of the paper was contacted to retrieve additional information.

### Statistical analysis

A meta-regression was used to quantify the association between HR of DFS and shortened time of adjuvant trastuzumab. Meta-regression is an extension of a standard meta-analysis that investigates associations between a treatment effect and a study characteristic^20^. The regression coefficient (*β*) is the estimated increase in the logarithmic HR per unit increase in the covariate. We used the “metareg” command in STATA, which was first introduced by Sharp and then modified by Knapp and Hartung^21^. The current “metareg” command included the modified features.

As Higgins et al.^22^ had demonstrated that standard meta-regression methods suffer from substantially inflated false-positive rates when heterogeneity is present, particularly in fixed effect meta-regression, we used a random-effects meta-regression model. The basic rationale of meta-regression consists of a weighted regression on a logarithmic scale between shortened treatment time and treatment effects on DFS. In this study, shortened treatment time (month) and DFS outcome (expressed as HRs) were extracted from each trial.

The default algorithm in “metareg” is restricted maximum likelihood to estimation of the between-study variance (*τ*^2^). The coefficient of determination (*R*^2^) was used to indicate the proportionate amount of variation in the response variable (expressed as ln[HR]) explained by the independent variables (expressed as shortened time) in the linear regression model. The larger the *R*^2^ value, the more variability is explained by the linear regression model.

Using the “metareg” command, we calculated the regression coefficient (*β*) of shortened time, indicating the change in the extent of ln(HR) of DFS when a given degree of shortened time changed. We estimated the regression equation to calculate the predicted HR based on regression coefficient (*β*) and shortened time: HR = *e*^(β* shortened time)^.

Sensitivity analysis were conducted by omitting each study to identify potential outliers. This analysis could be done using the “metainf” command^23^.

Publication bias was examined visually using the Begg’s funnel plot of ln(HR) against its SE and the degree of asymmetry was tested using the Egger’s test^14^.

Subgroup analysis were done according to the hormone receptor status (positive or negative), lymph node status (positive or negative), and chemotherapeutic regimens (anthracycline and taxane-containing chemotherapy or the others).

Two-sided *P* < 0.05 was considered statistically significant. All statistical analyses were performed using Stata v.13.0 (Stata Corporation, College Station, TX) and SPSS 17.0 (SPSS Inc, Chicago, IL).

## Supplementary material

Supplementary Figures

## Data Availability

The authors declare that all the data supporting the findings of this study are available within the paper.
