# Supplementary material for: Tailored duration of adjuvant trastuzumab for human epidermal growth factor receptor 2-positive breast cancer
Source: NPJ Precis Oncol. 2020 Aug 5;4:23. doi: 10.1038/s41698-020-00128-1 (PMC7406512; doi:10.1038/s41698-020-00128-1)

Supplemental Figures

Figure S1. Forest plot of overall meta-analysis

Forest plot of meta-analysis regarding the association of shorter duration of trastuzumab and breast cancer relapse. Center circles represent size of the hazard ratio (HR) estimated from each study. The 95% CIs for the HR are also shown. Overall HR is shown by the middle of a diamond whose left and right extremes represent the corresponding 95% CI. The heterogeneity P of 0.001 was not the P value for the pooled analysis.

Figure S1

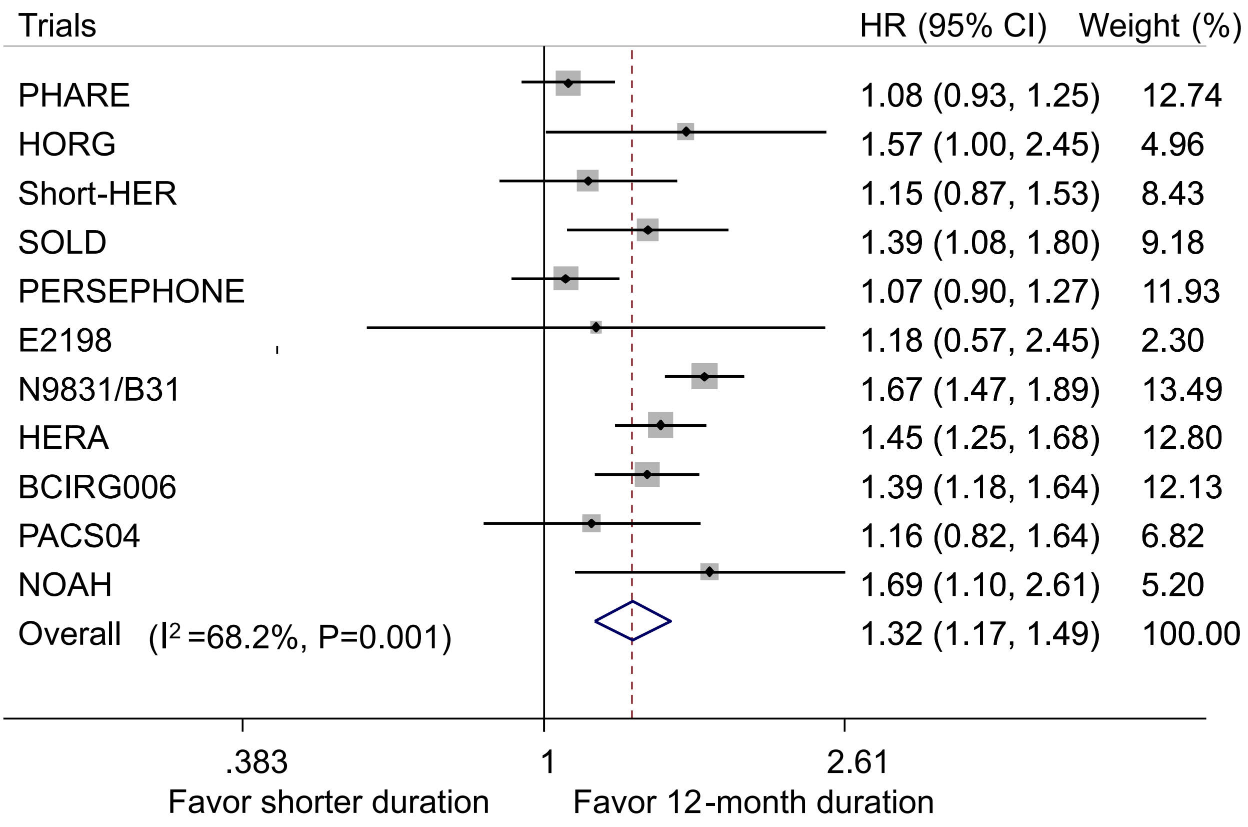

Figure S2. Begg's funnel plot of publication bias.

The vertical axis represents ln(HR) and the horizontal axis means the standard error of ln(HR). Horizontal line and sloping lines in funnel plot represent random-effect summary HR and expected 95% CI, respectively. Size of each circle represents contribution of the study to the overall effect.

Figure S2

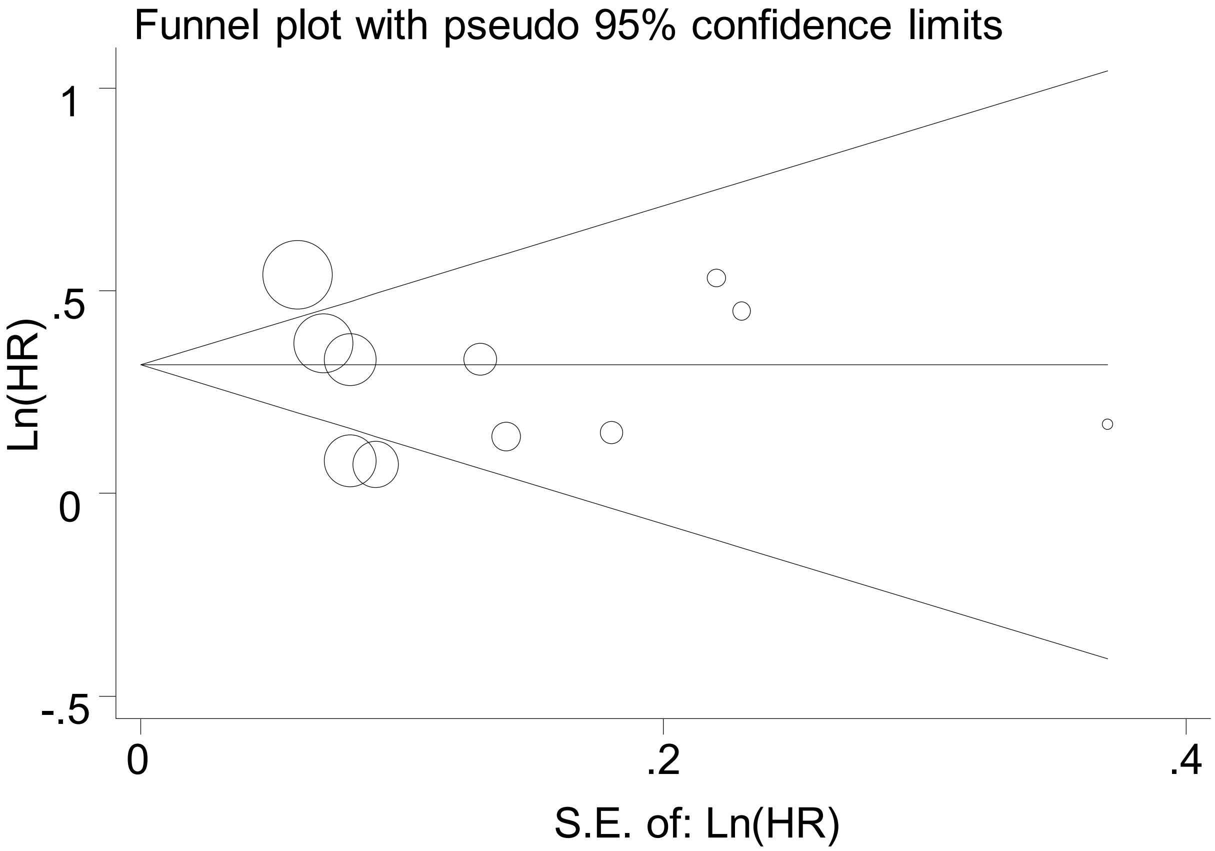

Supplement: Supplementary file 1 — Supplementary Figures [file 41698_2020_128_MOESM1_ESM.pdf]
